# Crystal structure of tris­[hexa­kis­(imidazole)cobalt(II)] bis­(benzene-1,3,5-tri­carboxyl­ate)

**DOI:** 10.1107/S2056989025010060

**Published:** 2025-11-25

**Authors:** Jose de Jesus Velazquez-Garcia, Christos Bintas, Faegheh Khademhir, Bassima Knjo, Aliyenur Ekineken, Fabienne Hain, Simone Techert

**Affiliations:** a Deutsches Elektronen-Synchrotron DESY Notkestr 85 22607 Hamburg Germany; b Department of Chemistry National and Kapodistrian University of Athens (NKUA) Zografou 157 72 Greece; c BS 06 Berufliche Schule Chemie, Biologie, Pharmazie, Agrarwirtschaft, Ladenbeker, Furtweg 151, 21033 Hamburg, Germany; d Institut für Röntgenphysik, Georg-August-Universität Göttingen, Friedrich-Hund-Platz 1, 37077 Göttingen, Germany; Universidad Nacional Autónoma de México, México

**Keywords:** crystal structure, trimesate, imidazole

## Abstract

The title compound, [Co^II^(C_3_H_4_N_2_)_6_]_3_(C_9_H_3_O_6_)_2_ (**1**), was synthesized from cobalt chloride(II), benzene-1,3,5-tri­carb­oxy­lic acid (H_3_btc) and imidazole (Im) in an ethanol/DMF mixture *via* slow evaporation at room temperature. This compound consists of three hexa­kis­(imidazole)­cobalt(II) cations and two trimesate anions. Examination of the crystal packing shows the formation of one-dimensional stacks of ions propagating along the *c* axis. The packing inter­actions are primarily driven by N—H⋯O hydrogen bonding between anions and cations.

## Abstract 2

The structure of a tris­[hexa­kis­(imidazole)­cobalt(II)] bis­(benzene-1,3,5-tri­carboxyl­ate) compound was determined by single-crystal X-ray diffraction.

## 1. Chemical context

Benzene-1,3,5-tri­carb­oxy­lic acid (trimesic acid, H_3_btc) and imidazole derivatives are typically used in the synthesis of metal–organic frameworks (MOFs). For example, imidazole (Im) and 2-methyl­imidazole (2mIm) serve as ligands in the synthesis of the reported zeolitic imidazolate frameworks ZIF-4 and ZIF-8, respectively (Park *et al.*, 2006 ▸). Likewise, H_3_btc is a key precursor in the synthesis of the well-known MOFs MIL-100 (Férey *et al.*, 2004 ▸) and HKUST-1 (Chui *et al.*, 1999 ▸). In previous work, we have employed 2-methyl­imidazole and H_3_btc to synthesize a small coordination complex (Velazquez-Garcia & Techert, 2022 ▸), various organic salts (Baletska *et al.*, 2023 ▸; Asprilla-Herrera *et al.*, 2025 ▸; Łukaszczyk *et al.*, 2025 ▸) and two mixed-ligand MOFs (Velazquez Garcia *et al.*, 2025 ▸). In the present study, we substituted 2-methyl­imidazole with imidazole to synthesize the title compound (**1**).
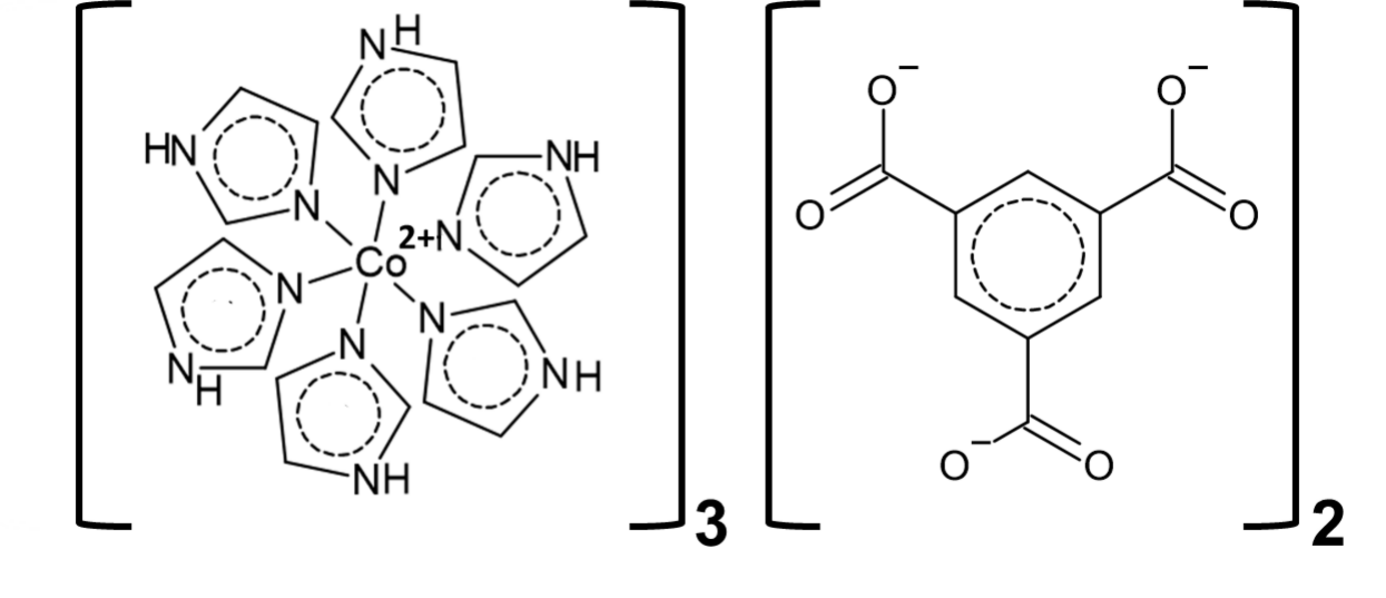


## 2. Structural commentary

**Figure 1:**
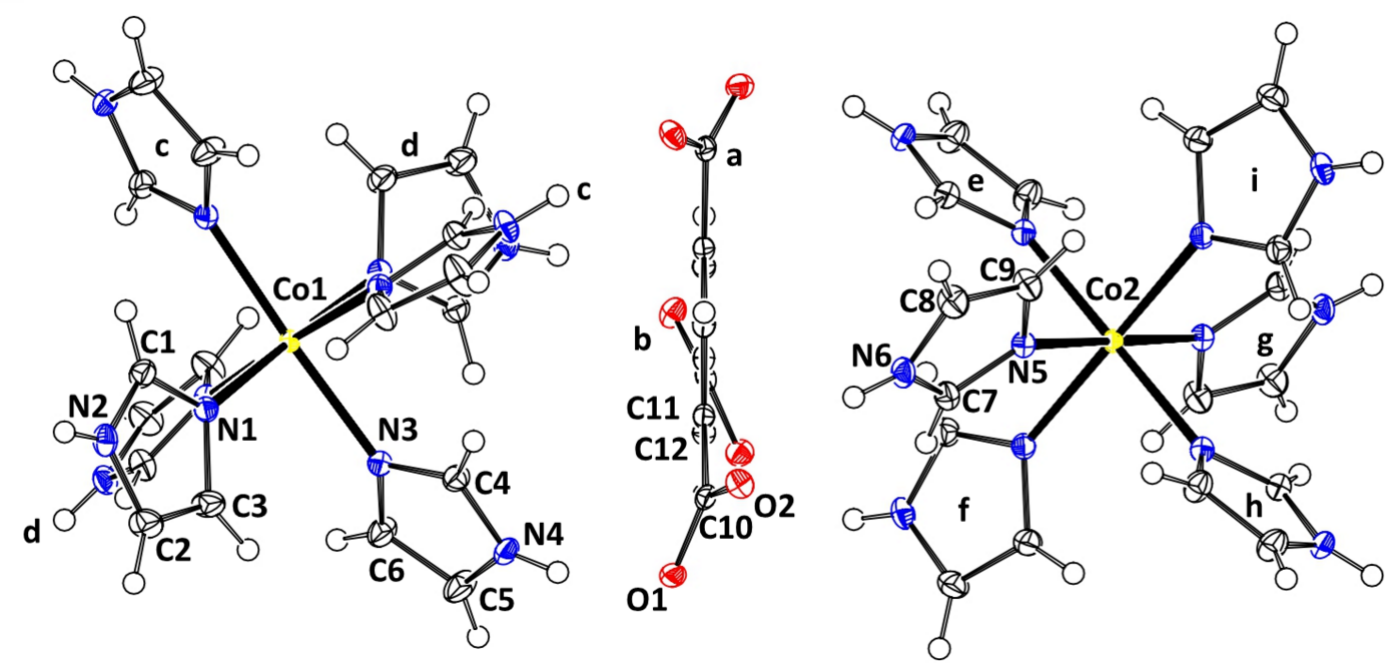
Crystal structure of **1** with displacement ellipsoids drawn at the 50% probability level. Atoms labelled by group are generated by the following symmetry operations: 1 − *y*, 1 + *x* − *y*, *z* for groups a, c and e; −*x* + *y*, 1 − *x*, *z* for groups b, d and f; 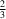 − *x*, 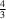 − *y*, 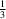 − *z* for fractions g and i; −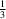 + *y*, 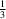 − *x* + *y*, 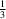 − *z* for group h.

Compound **1** (Fig. 1 ▸) crystallizes in the trigonal *R*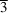 space group. The complete group contains three hexa­kis­(imidazole)­cobalt(II) cations and two fully deprotonated btc^3−^ anions. The asymmetric unit comprises one third of a btc^3−^ anion and two crystallographically independent metal centres (Co1 and Co2) – one third of Co1 coordinated by two Im ligands and one sixth of Co2 coordinated by a single Im ligand. Both metal centres and the centre of mass of the btc^3−^ ion lie on the 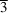 rotoinversion axis, while Co2 is located exactly on the inversion centre.

To estimate the distortion from the ideal octa­hedral geometry of the cations, the parameters Σ (Halcrow, 2011 ▸) and Θ (Marchivie *et al.*, 2005 ▸) were calculated using the *OctaDist* program (Ketkaew *et al.*, 2021 ▸). While Σ summarizes the deviation of the N—Co—N angles from 90°, Θ indicates the degree of twist from a perfect octa­hedron towards a trigonal prism. In an ideal octa­hedron, both parameters are equal to zero, whereas Θ reaches 1140° for a perfect trigonal prism. The calculated values of the distortion parameters Σ/Θ for Co1 and Co2 are equal to 13°/27° and 10°/23°, respectively. Both parameters exhibit a slight distortion of the coordination environment of both metal centres.

## 3. Supra­molecular features

**Figure 2:**
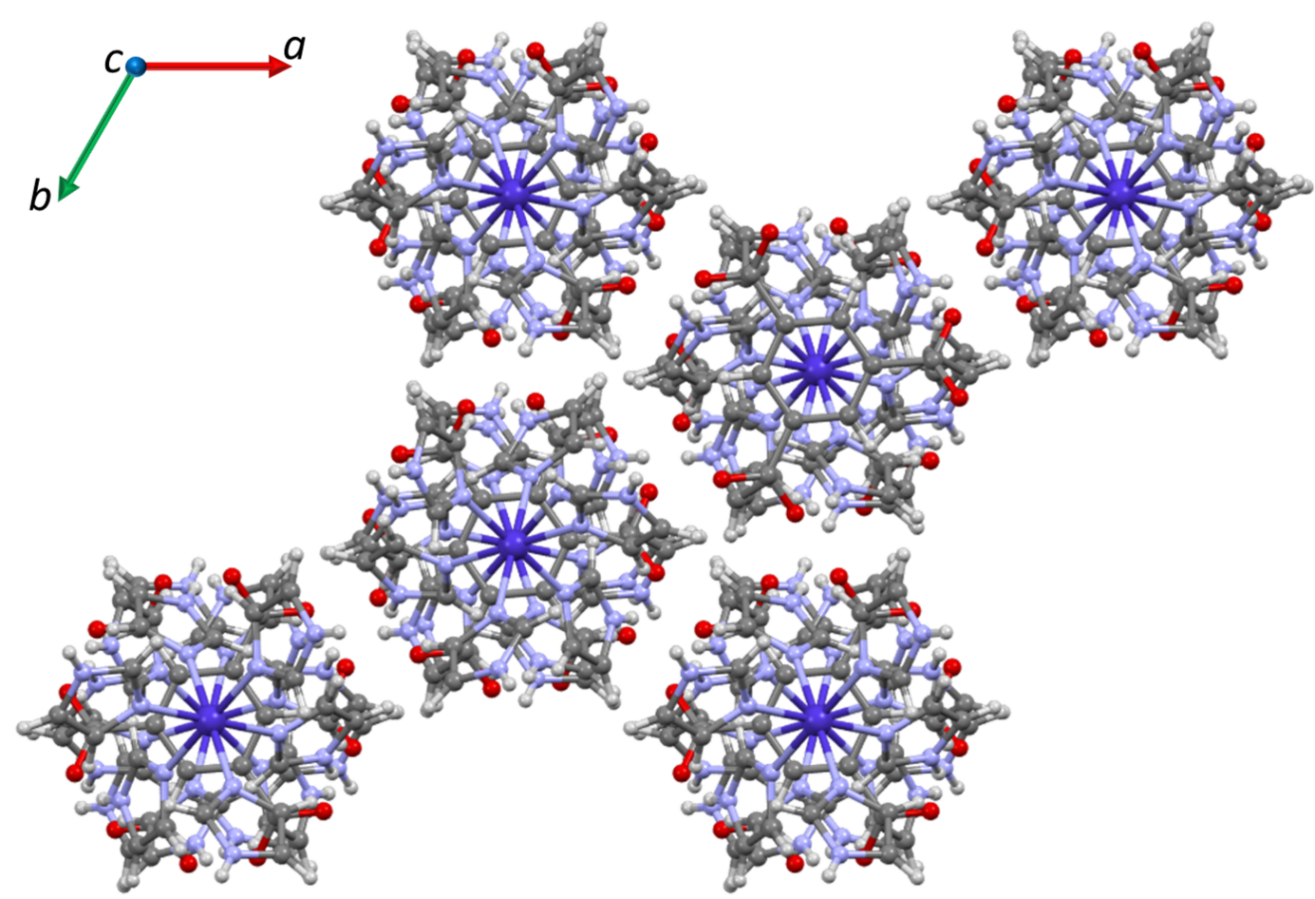
Packing diagram of **1** down the *c* axis.

**Figure 3:**
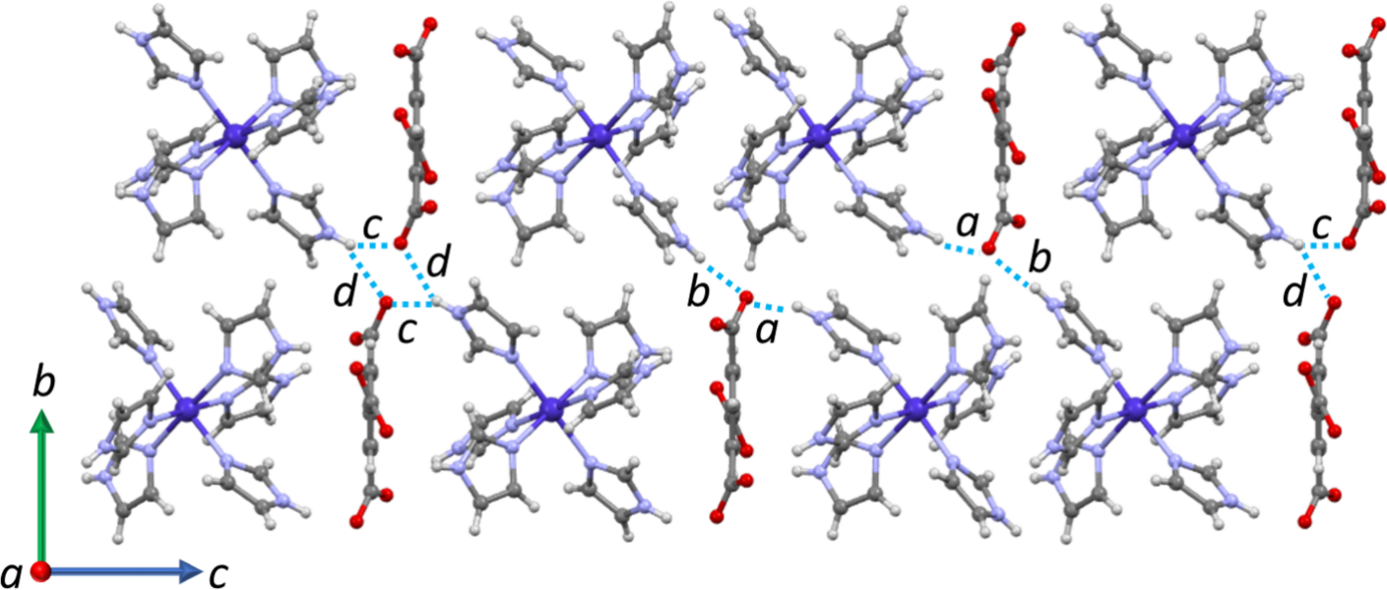
Crystal packing of **1** viewed along the *a* axis, showing only two representative stacks for clarity. Only discrete graph-set motifs are highlighted (*a*–*d*).

Crystal packing diagrams of compound **1** as viewed down the *c* and *a* axes are shown in Figs. 2 ▸ and 3 ▸, respectively. The figures show columns of ions stacked along the *c* axis, following a repeating polar arrangement: anion – cation – cation – anion – cation. Each column inter­acts with others *via* hydrogen bonding of the N—H⋯O type (Fig. 3 ▸), summarized in Table 1 ▸. The table demonstrates that all possible donor and acceptor groups are involved in moderate hydrogen bonds. The presence of different hydrogen bonds in **1** results in characteristic arrays that may be described by graph-set analysis (Etter *et al.*, 1990 ▸; Bernstein *et al.*, 1995 ▸)**.** In the structure of **1**, there are ten motifs involved in discrete *D* (all types), ring *R* (all types) and chains *C* (types *b* and *d*). Notably, hydrogen bonds *b* and *d* hold the aforementioned columns together, whereas *a* and *c* strengthen the inter­action between ions within the columns.

## 4. Database survey

No reported structures of the title compound were found in the Cambridge Structural Database (CSD version 5.45, update of November 2023; Groom *et al.*, 2016 ▸). Some structures containing the hexa­kis­(imidazole)­cobalt(II) cation and polycarboxyl­ate anions were reported under the refcodes AGAXIS (Jyai & Srinivasan, 2019 ▸), BOVMIJ (Nie *et al.*, 2009 ▸) and EFIVOE (Tong *et al.*, 2002 ▸). However, none of them include btc^3−^ as counter-ion but benzene-1,2-dicarboxylate, bis­(naphthalene-1,4-di­carboxyl­ate) and 1,4-benzene­dicarb­oxyl­ate, respectively.

## 5. Synthesis and crystallization

In a typical synthesis, 100 µL of a 0.11 *M* ethano­lic solution of CoCl_2_·6H_2_O was diluted with 100 µL of *N*,*N*-di­methyl­formamide, followed by the addition of 120 µL of a 1.58 *M* ethano­lic solution of imidazole. Then, 100 µL of a 0.12 *M* ethano­lic solution of H_3_btc was added to the mixture. The resulting mixture was gently shaken and allowed to dry slowly at room temperature. After three weeks, red crystals of **1** were obtained.

## 6. Refinement

Crystal data, data collection and structure refinement details are summarized in Table 2 ▸. The positions of hydrogen atoms were refined with *U*_iso_(H) = 1.2*U*_eq_(C or N) for CH and NH groups. Hydrogen atoms bonded to nitro­gen atoms (N—H) were treated with free refinement of bond distances. The most disagreeable reflections (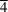
50 and 244) with error/s.u. of more than ten were omitted using the OMIT instruction in *SHELXL* (Sheldrick, 2015*b* ▸).

## Supplementary Material

Crystal structure: contains datablock(s) I. DOI: 10.1107/S2056989025010060/jq2041sup1.cif

Structure factors: contains datablock(s) I. DOI: 10.1107/S2056989025010060/jq2041Isup2.hkl

CCDC reference: 2502103

Additional supporting information:  crystallographic information; 3D view; checkCIF report

## Figures and Tables

**Table 1:** Hydrogen-bond geometry (Å,°)

*D*—H⋯*A*	Type	Graph-set	*D*—H	H⋯*A*	*D*⋯*A*	*D*—H⋯*A*
N4—H4⋯O1	*a*	*DR*^2^_2_(16)	0.856 (14)	1.908 (14)	2.7282 (17)	160.0 (4)
N2—H2⋯O1^i^	*b*	*DC*^2^_2_(16) 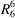 (48)	0.835 (13)	1.991 (13)	2.7661 (18)	154.0 (12)
N6—H6*A*⋯O2	*c*	*DR*^2^_2_(16)	0.841 (14)	2.075 (12)	2.8161 (17)	146.8 (4)
N6—H6*A*⋯O2^ii^	*d*	*DC*^2^_2_(16) 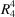 (32) 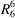 (48)	0.841 (14)	2.446 (9)	3.0298 (17)	127.2 (3)

Symmetry codes: (i) 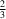 − *x* + *y*, 4/3 − *x*, 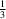 + *z*; (ii) 4/3 − *x*, 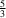 − *y*, 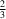 − *z*.

**Table 2:** Experimental details

Crystal data	
Chemical formula	[Co(C_3_H_4_N_2_)_6_]_3_(C_9_H_3_O_6_)_2_
*M*_r_	1816.49
Crystal system, space group	Trigonal, *R* 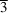
Temperature (K)	100
*a*, *c* (Å)	15.215 (2), 30.494 (6)
*V* (Å^3^)	6113 (2)
*Z*	3
Radiation type	Mo *K*α
μ (mm^−1^)	0.69
Crystal size (mm)	0.6 × 0.4 × 0.2

Data collection	
Diffractometer	Bruker P4
Absorption correction	Multi-scan (*SADABS*; Krause *et al.*, 2015 ▸)
*T*_min_, *T*_max_	0.690, 0.748
No. of measured, independent and observed [*I* > 2σ(*I*)] reflections	28365, 3380, 3106
*R*_int_	0.031
(sin θ/λ)_max_ (Å^−1^)	0.667

Refinement	
*R*[*F*^2^ > 2σ(*F*^2^)], *wR*(*F*^2^), *S*	0.028, 0.076, 1.04
No. of reflections	3380
No. of parameters	199
H-atom treatment	H-atom parameters constrained
Δρ_max_, Δρ_min_ (e Å^−3^)	0.45, −0.33

Computer programs: *APEX2* and *APEX2* (Bruker, 2016 ▸), *SHELXT2018/2* (Sheldrick, 2015*a* ▸), *SHELXL2018/3* (Sheldrick, 2015*b* ▸) and *OLEX2* (Dolomanov *et al.*, 2009 ▸).

## supplementary crystallographic information

### Tris[hexakis(imidazole)cobalt(II)] bis(benzene-1,3,5-tricarboxylate) Crystal data

**Table:**

[Co(C_3_H_4_N_2_)_6_]_3_(C_9_H_3_O_6_)_2_	*D*_x_ = 1.480 Mg m^−^^3^
*M**_r_* = 1816.49	Mo *K*α radiation, λ = 0.71073 Å
Trigonal, *R*3	Cell parameters from 9869 reflections
*a* = 15.215 (2) Å	θ = 2.7–39.6°
*c* = 30.494 (6) Å	µ = 0.69 mm^−^^1^
*V* = 6113 (2) Å^3^	*T* = 100 K
*Z* = 3	Rhombohedral, red
*F*(000) = 2817	0.6 × 0.4 × 0.2 mm

### Tris[hexakis(imidazole)cobalt(II)] bis(benzene-1,3,5-tricarboxylate) Data collection

**Table:**

Bruker P4 diffractometer	*R*_int_ = 0.031
ω scans	θ_max_ = 28.3°, θ_min_ = 1.7°
Absorption correction: multi-scan (SADABS; Krause *et al.*, 2015)	*h* = −20→20
*T*_min_ = 0.690, *T*_max_ = 0.748	*k* = −20→20
28365 measured reflections	*l* = −40→40
3380 independent reflections	Standard reflections: not measured; every not measured reflections
3106 reflections with *I* > 2σ(*I*)	intensity decay: not measured

### Tris[hexakis(imidazole)cobalt(II)] bis(benzene-1,3,5-tricarboxylate) Refinement

**Table:**

Refinement on *F*^2^	Primary atom site location: dual
Least-squares matrix: full	Hydrogen site location: inferred from neighbouring sites
*R*[*F*^2^ > 2σ(*F*^2^)] = 0.028	H-atom parameters constrained
*wR*(*F*^2^) = 0.076	*w* = 1/[σ^2^(*F*_o_^2^) + (0.0345*P*)^2^ + 12.840*P*] where *P* = (*F*_o_^2^ + 2*F*_c_^2^)/3
*S* = 1.04	(Δ/σ)_max_ = 0.001
3380 reflections	Δρ_max_ = 0.45 e Å^−^^3^
199 parameters	Δρ_min_ = −0.32 e Å^−^^3^
0 restraints	

### Tris[hexakis(imidazole)cobalt(II)] bis(benzene-1,3,5-tricarboxylate) Special details

**Table:**

Geometry. All esds (except the esd in the dihedral angle between two l.s. planes) are estimated using the full covariance matrix. The cell esds are taken into account individually in the estimation of esds in distances, angles and torsion angles; correlations between esds in cell parameters are only used when they are defined by crystal symmetry. An approximate (isotropic) treatment of cell esds is used for estimating esds involving l.s. planes.

### Tris[hexakis(imidazole)cobalt(II)] bis(benzene-1,3,5-tricarboxylate) Fractional atomic coordinates and isotropic or equivalent isotropic displacement parameters (Å^2^)

**Table:**

	*x*	*y*	*z*	*U*_iso_*/*U*_eq_	
Co1	0.333333	0.666667	0.55111 (2)	0.00919 (8)	
Co2	0.333333	0.666667	0.166667	0.00965 (10)	
O2	0.59402 (7)	0.71916 (7)	0.34095 (3)	0.01540 (19)	
O1	0.50598 (7)	0.56614 (7)	0.37221 (3)	0.01563 (19)	
N1	0.46175 (8)	0.69448 (8)	0.59020 (3)	0.0127 (2)	
N3	0.35884 (8)	0.56677 (8)	0.50903 (3)	0.0129 (2)	
N5	0.46325 (8)	0.75844 (8)	0.20837 (3)	0.0131 (2)	
N4	0.40359 (9)	0.51431 (9)	0.44997 (4)	0.0165 (2)	
H4	0.4338 (7)	0.51618 (10)	0.4260 (5)	0.020*	
N2	0.58846 (8)	0.75732 (9)	0.63733 (4)	0.0160 (2)	
H2	0.6292 (9)	0.7952 (9)	0.6564 (4)	0.019*	
N6	0.56893 (9)	0.80854 (9)	0.26463 (4)	0.0163 (2)	
H6A	0.5978 (7)	0.80585 (11)	0.2877 (5)	0.020*	
C11	0.42044 (9)	0.65755 (9)	0.35769 (4)	0.0121 (2)	
C12	0.32434 (9)	0.57106 (9)	0.35767 (4)	0.0128 (2)	
H12	0.31828 (15)	0.5068 (12)	0.35765 (4)	0.015*	
C10	0.51488 (9)	0.64817 (9)	0.35684 (4)	0.0122 (2)	
C4	0.41613 (10)	0.59442 (10)	0.47339 (4)	0.0153 (2)	
H4A	0.4609 (9)	0.6629 (13)	0.46533 (16)	0.018*	
C7	0.49165 (10)	0.72946 (10)	0.24412 (4)	0.0152 (2)	
H7	0.4615 (6)	0.6621 (13)	0.25389 (19)	0.018*	
C1	0.51171 (10)	0.76558 (10)	0.61989 (4)	0.0170 (2)	
H1	0.4959 (3)	0.8149 (10)	0.62771 (16)	0.020*	
C9	0.52659 (10)	0.86245 (10)	0.20645 (4)	0.0184 (3)	
H9	0.52491 (11)	0.9039 (9)	0.1851 (4)	0.022*	
C8	0.59212 (11)	0.89424 (11)	0.24123 (5)	0.0202 (3)	
H8	0.6424 (10)	0.9607 (14)	0.24769 (14)	0.024*	
C2	0.58792 (11)	0.67609 (11)	0.61821 (5)	0.0211 (3)	
H2A	0.6330 (9)	0.6514 (5)	0.62393 (13)	0.025*	
C3	0.50915 (11)	0.63748 (11)	0.58913 (5)	0.0201 (3)	
H3	0.4896 (4)	0.5789 (12)	0.5706 (4)	0.024*	
C6	0.30649 (11)	0.46203 (10)	0.50802 (4)	0.0200 (3)	
H6	0.2597 (10)	0.4201 (9)	0.5291 (4)	0.024*	
C5	0.33334 (12)	0.42898 (11)	0.47158 (5)	0.0243 (3)	
H5	0.3089 (5)	0.3622 (15)	0.46314 (19)	0.029*	

### Tris[hexakis(imidazole)cobalt(II)] bis(benzene-1,3,5-tricarboxylate) Atomic displacement parameters (Å^2^)

**Table:**

	*U*^11^	*U*^22^	*U*^33^	*U*^12^	*U*^13^	*U*^23^
Co1	0.00977 (10)	0.00977 (10)	0.00803 (13)	0.00488 (5)	0.000	0.000
Co2	0.01060 (13)	0.01060 (13)	0.00775 (18)	0.00530 (6)	0.000	0.000
O2	0.0124 (4)	0.0163 (4)	0.0162 (4)	0.0061 (4)	−0.0005 (3)	0.0012 (3)
O1	0.0194 (4)	0.0169 (4)	0.0148 (4)	0.0122 (4)	0.0048 (3)	0.0028 (3)
N1	0.0134 (5)	0.0136 (5)	0.0114 (5)	0.0069 (4)	0.0001 (4)	0.0005 (4)
N3	0.0138 (5)	0.0136 (5)	0.0121 (5)	0.0074 (4)	−0.0003 (4)	−0.0004 (4)
N5	0.0135 (5)	0.0142 (5)	0.0114 (5)	0.0067 (4)	−0.0001 (4)	−0.0002 (4)
N4	0.0216 (6)	0.0174 (5)	0.0128 (5)	0.0116 (5)	0.0047 (4)	0.0000 (4)
N2	0.0133 (5)	0.0196 (5)	0.0125 (5)	0.0062 (4)	−0.0026 (4)	−0.0019 (4)
N6	0.0161 (5)	0.0218 (6)	0.0118 (5)	0.0101 (5)	−0.0037 (4)	−0.0026 (4)
C11	0.0132 (5)	0.0151 (6)	0.0093 (5)	0.0080 (5)	0.0003 (4)	0.0000 (4)
C12	0.0153 (6)	0.0127 (6)	0.0109 (5)	0.0074 (5)	−0.0005 (4)	−0.0003 (4)
C10	0.0139 (5)	0.0150 (6)	0.0091 (5)	0.0082 (5)	−0.0012 (4)	−0.0030 (4)
C4	0.0167 (6)	0.0143 (6)	0.0151 (6)	0.0079 (5)	0.0023 (5)	0.0002 (5)
C7	0.0150 (6)	0.0174 (6)	0.0131 (6)	0.0080 (5)	−0.0008 (4)	0.0003 (4)
C1	0.0175 (6)	0.0205 (6)	0.0148 (6)	0.0109 (5)	−0.0021 (5)	−0.0039 (5)
C9	0.0191 (6)	0.0149 (6)	0.0171 (6)	0.0053 (5)	−0.0035 (5)	0.0009 (5)
C8	0.0190 (6)	0.0166 (6)	0.0204 (6)	0.0055 (5)	−0.0053 (5)	−0.0022 (5)
C2	0.0182 (6)	0.0241 (7)	0.0246 (7)	0.0133 (6)	−0.0062 (5)	−0.0036 (5)
C3	0.0201 (6)	0.0211 (7)	0.0238 (7)	0.0138 (6)	−0.0074 (5)	−0.0060 (5)
C6	0.0262 (7)	0.0140 (6)	0.0179 (6)	0.0087 (5)	0.0081 (5)	0.0017 (5)
C5	0.0334 (8)	0.0139 (6)	0.0228 (7)	0.0098 (6)	0.0106 (6)	0.0000 (5)

### Tris[hexakis(imidazole)cobalt(II)] bis(benzene-1,3,5-tricarboxylate) Geometric parameters (Å, º)

**Table:**

Co1—N1	2.1426 (11)	N2—H2	0.836 (19)
Co1—N1^i^	2.1426 (11)	N2—C1	1.3448 (17)
Co1—N1^ii^	2.1426 (11)	N2—C2	1.3629 (18)
Co1—N3	2.1673 (11)	N6—H6A	0.840 (19)
Co1—N3^ii^	2.1673 (11)	N6—C7	1.3441 (17)
Co1—N3^i^	2.1673 (11)	N6—C8	1.3689 (18)
Co2—N5^iii^	2.1710 (11)	C11—C12^i^	1.3965 (17)
Co2—N5^ii^	2.1712 (11)	C11—C12	1.3948 (17)
Co2—N5	2.1711 (11)	C11—C10	1.5136 (17)
Co2—N5^iv^	2.1711 (11)	C12—H12	0.935 (18)
Co2—N5^i^	2.1711 (11)	C4—H4A	0.949 (18)
Co2—N5^v^	2.1711 (11)	C7—H7	0.938 (18)
O2—C10	1.2452 (16)	C1—H1	0.927 (18)
O1—C10	1.2755 (15)	C9—H9	0.917 (19)
N1—C1	1.3212 (17)	C9—C8	1.3679 (18)
N1—C3	1.3780 (17)	C8—H8	0.935 (19)
N3—C4	1.3233 (16)	C2—H2A	0.948 (19)
N3—C6	1.3805 (17)	C2—C3	1.3652 (19)
N5—C7	1.3269 (16)	C3—H3	0.968 (19)
N5—C9	1.3826 (17)	C6—H6	0.935 (19)
N4—H4	0.855 (18)	C6—C5	1.3637 (19)
N4—C4	1.3413 (17)	C5—H5	0.93 (2)
N4—C5	1.3690 (18)		
			
N1—Co1—N1^i^	92.04 (4)	C1—N2—H2	126.2
N1—Co1—N1^ii^	92.04 (4)	C1—N2—C2	107.69 (11)
N1^i^—Co1—N1^ii^	92.04 (4)	C2—N2—H2	126.2
N1^i^—Co1—N3^ii^	89.24 (4)	C7—N6—H6A	126.2
N1^ii^—Co1—N3^i^	177.42 (4)	C7—N6—C8	107.68 (11)
N1—Co1—N3^i^	89.24 (4)	C8—N6—H6A	126.2
N1^ii^—Co1—N3^ii^	90.15 (4)	C12—C11—C12^i^	119.40 (12)
N1—Co1—N3	90.15 (4)	C12—C11—C10	120.52 (11)
N1^i^—Co1—N3^i^	90.15 (4)	C12^i^—C11—C10	120.07 (11)
N1^i^—Co1—N3	177.42 (4)	C11—C12—C11^ii^	120.60 (12)
N1^ii^—Co1—N3	89.24 (4)	C11—C12—H12	119.7
N1—Co1—N3^ii^	177.42 (4)	C11^ii^—C12—H12	119.7
N3—Co1—N3^i^	88.52 (4)	O2—C10—O1	125.12 (11)
N3—Co1—N3^ii^	88.52 (4)	O2—C10—C11	118.48 (11)
N3^ii^—Co1—N3^i^	88.52 (4)	O1—C10—C11	116.40 (11)
N5^iii^—Co2—N5^iv^	89.17 (4)	N3—C4—N4	112.12 (12)
N5^v^—Co2—N5	90.83 (4)	N3—C4—H4A	123.9
N5^v^—Co2—N5^ii^	180.00 (6)	N4—C4—H4A	123.9
N5^iii^—Co2—N5^i^	180.0	N5—C7—N6	111.68 (12)
N5^iv^—Co2—N5^ii^	90.83 (4)	N5—C7—H7	124.2
N5^iv^—Co2—N5^i^	90.83 (4)	N6—C7—H7	124.2
N5^i^—Co2—N5	89.17 (4)	N1—C1—N2	111.44 (12)
N5^iii^—Co2—N5^v^	89.17 (4)	N1—C1—H1	124.3
N5^iii^—Co2—N5^ii^	90.83 (4)	N2—C1—H1	124.3
N5^iv^—Co2—N5^v^	89.17 (4)	N5—C9—H9	125.1
N5^i^—Co2—N5^ii^	89.17 (4)	C8—C9—N5	109.85 (12)
N5^iii^—Co2—N5	90.83 (4)	C8—C9—H9	125.1
N5—Co2—N5^ii^	89.17 (4)	N6—C8—H8	127.1
N5^iv^—Co2—N5	180.00 (4)	C9—C8—N6	105.80 (12)
N5^i^—Co2—N5^v^	90.83 (4)	C9—C8—H8	127.1
C1—N1—Co1	129.61 (9)	N2—C2—H2A	127.1
C1—N1—C3	105.28 (11)	N2—C2—C3	105.89 (12)
C3—N1—Co1	125.11 (9)	C3—C2—H2A	127.1
C4—N3—Co1	125.72 (9)	N1—C3—H3	125.2
C4—N3—C6	104.88 (11)	C2—C3—N1	109.70 (12)
C6—N3—Co1	128.35 (9)	C2—C3—H3	125.2
C7—N5—Co2	127.63 (9)	N3—C6—H6	125.1
C7—N5—C9	105.00 (11)	C5—C6—N3	109.72 (12)
C9—N5—Co2	126.92 (9)	C5—C6—H6	125.1
C4—N4—H4	126.4	N4—C5—H5	126.9
C4—N4—C5	107.12 (11)	C6—C5—N4	106.16 (12)
C5—N4—H4	126.4	C6—C5—H5	126.9
			
Co1—N1—C1—N2	179.95 (8)	C10—C11—C12—C11^ii^	−178.88 (8)
Co1—N1—C3—C2	−179.87 (10)	C4—N3—C6—C5	−0.21 (16)
Co1—N3—C4—N4	−169.11 (8)	C4—N4—C5—C6	−0.41 (17)
Co1—N3—C6—C5	168.45 (10)	C7—N5—C9—C8	−0.03 (16)
Co2—N5—C7—N6	−172.93 (8)	C7—N6—C8—C9	−0.42 (15)
Co2—N5—C9—C8	172.72 (9)	C1—N1—C3—C2	−0.46 (16)
N3—C6—C5—N4	0.39 (18)	C1—N2—C2—C3	0.16 (16)
N5—C9—C8—N6	0.28 (16)	C9—N5—C7—N6	−0.24 (15)
N2—C2—C3—N1	0.18 (17)	C8—N6—C7—N5	0.42 (15)
C12^i^—C11—C12—C11^ii^	−0.1 (2)	C2—N2—C1—N1	−0.47 (16)
C12^i^—C11—C10—O2	−25.08 (17)	C3—N1—C1—N2	0.57 (15)
C12—C11—C10—O2	153.71 (12)	C6—N3—C4—N4	−0.05 (15)
C12—C11—C10—O1	−25.38 (17)	C5—N4—C4—N3	0.30 (16)
C12^i^—C11—C10—O1	155.82 (11)		

Symmetry codes: (i) −*y*+1, *x*−*y*+1, *z*; (ii) −*x*+*y*, −*x*+1, *z*; (iii) *y*−1/3, −*x*+*y*+1/3, −*z*+1/3; (iv) −*x*+2/3, −*y*+4/3, −*z*+1/3; (v) *x*−*y*+2/3, *x*+1/3, −*z*+1/3.
